# Toward the Selection of Cell Targeting Aptamers with Extended Biological Functionalities to Facilitate Endosomal Escape of Cargoes

**DOI:** 10.3390/biomedicines5030051

**Published:** 2017-08-24

**Authors:** Kwaku D. Tawiah, David Porciani, Donald H. Burke

**Affiliations:** 1 Department of Biochemistry, University of Missouri, Columbia, MO 65211, USA; kdtkc3@mail.missouri.edu; 2 Bond Life Sciences Center, University of Missouri, Columbia, MO 65211, USA; 3 Department of Molecular Microbiology & Immunology, University of Missouri, Columbia, MO 65212, USA; 4 Department of Bioengineering, University of Missouri, Columbia, MO 65211, USA

**Keywords:** aptamers, targeted drug delivery, endosomal escape, SELEX, cancer therapy

## Abstract

Over the past decades there have been exciting and rapid developments of highly specific molecules to bind cancer antigens that are overexpressed on the surfaces of malignant cells. Nanomedicine aims to exploit these ligands to generate nanoscale platforms for targeted cancer therapy, and to do so with negligible off-target effects. Aptamers are structured nucleic acids that bind to defined molecular targets ranging from small molecules and proteins to whole cells or viruses. They are selected through an iterative process of amplification and enrichment called SELEX (systematic evolution of ligands by exponential enrichment), in which a combinatorial oligonucleotide library is exposed to the target of interest for several repetitive rounds. Nucleic acid ligands able to bind and internalize into malignant cells have been extensively used as tools for targeted delivery of therapeutic payloads both in vitro and in vivo. However, current cell targeting aptamer platforms suffer from limitations that have slowed their translation to the clinic. This is especially true for applications in which the cargo must reach the cytosol to exert its biological activity, as only a small percentage of the endocytosed cargo is typically able to translocate into the cytosol. Innovative technologies and selection strategies are required to enhance cytoplasmic delivery. In this review, we describe current selection methods used to generate aptamers that target cancer cells, and we highlight some of the factors that affect productive endosomal escape of cargoes. We also give an overview of the most promising strategies utilized to improve and monitor endosomal escape of therapeutic cargoes. The methods we highlight exploit tools and technologies that can potentially be incorporated in the SELEX process. Innovative selection protocols may identify aptamers with extended biological functionalities that allow effective cytosolic translocation of therapeutics. This in turn may facilitate successful translation of these platforms into clinical applications.

## 1. Introduction

Cancer remains one of the leading causes of death worldwide, with high morbidity and mortality. Though significant advances have been made in the fundamental understanding of cancer biology, the treatment of some cancer forms remains elusive, and many cancer treatments suffer from severe side effects [1,2]. Over the past decades, novel active biomolecules such as regulatory peptides or short interfering RNAs are emerging as promising agents for cancer therapy and for studying tissue-specific gene function [3,4,5]. However, they possess some limitations. In particular, siRNAs and most regulatory peptides are relatively large, highly polar and charged molecules that require delivery vehicles to cross the plasma membrane. Promising delivery strategies have been developed in the form of lipid and polymeric nanoparticles [6,7]. However, these strategies usually lack selectivity and are unable to discriminate between tumor and healthy cells, causing off-target effects [8]. In light of these considerations, significant efforts have been devoted to developing effective targeted delivery nanostructures able to direct these therapeutics specifically toward cancer cells [8,9]. A common strategy to enhance selectivity relies on developing ligands that bind biomolecules that are overexpressed on the surfaces of malignant cells. To date, the most used recognition tools are proteins (antibodies and their fragments) [10,11], peptides [11], oligonucleotides (nucleic acid aptamers) [12], and small molecules (vitamins, carbohydrates) [13]. Several prior reviews have highlighted the structural requirements (size and charge) and pharmacokinetic features that a ligand-drug platform should possess to minimize side effects and increase its therapeutic window [2,8]. However, only a few studies have performed a side-by-side analysis in vitro and in vivo to compare selectivity of these classes of ligands [14].

Aptamers are short, single-chained DNA or RNA molecules that recognize molecular targets with high affinity (in nM to pM range) and specificity owing to their 3D conformation. Aptamers are emerging as a class of biocompatible ligands with huge potential in diagnostics and therapeutics [15,16,17]. Compared with antibodies, aptamer production exploits a chemical technology with high batch fidelity that is not intrinsically prone to viral or bacterial contamination [15]. In addition, the process of chemical synthesis allows the site-specific introduction of non-nucleotide linkers (e.g., hexaethylene glycol), or chemical functionalities useful for conjugation (such as primary amines, thiols, and terminal alkynes), and the addition of fluorescent or other reporter moieties. Moreover, due to their small sizes (aptamers: 25–70 nt equals 8–20 kDa; common antibodies ~150–160 kDa; Fab fragments ~50 kDa; and single chain variable fragments ~25 kDa) [1], aptamers can penetrate into biological compartments more efficiently than common antibodies and most of the engineered antibody fragments, and they can potentially access protein structural domains that might be sterically inaccessible to bulkier ligands [15].

**Figure 1:**
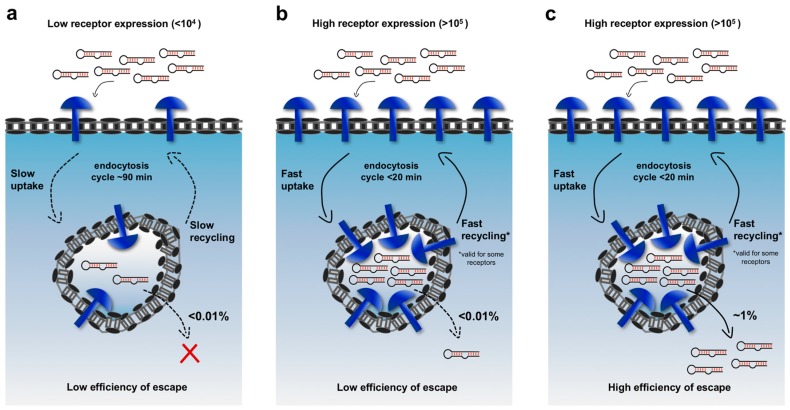
Relationships among three key factors that govern cytosolic delivery: receptor expression on the cell surface, rate of endocytosis and endosomal escape efficiency of cargoes. In (**a**,**b**) the estimated efficiency of escape is <0.01% per cycle of endocytosis; (**a**) Upon binding to low-expressed receptors (<10^4^ copies/cell) that internalize with slow rate of uptake (~90 min), a negligible cytoplasmic translocation of internalized aptamers occurs; (**b**) Increased translocation can be observed when cell targeting aptamers bind highly expressed receptors (>10^5^ copies/cell) that show faster internalization (~20 min). This situation can, in some extent, resemble some successful cancer therapy strategies aim to target asialoglycoprotein receptor (ASGPR), which is expressed at high levels on the surface of liver cells (**c**) An ideal delivery system should bind highly expressed receptors (>10^5^) with high rate of uptake (~20 min) and should possess an enhanced endosomal escape efficiency (i.e., high efficiency of cargoes ~1%/cycle).

Despite the rapid development of diverse platforms that exploit aptamers to cell-surface receptors as vehicles to deliver peptides and siRNAs, none has made it into clinical trials yet [15]. Some of the translational bottlenecks are related to current technological limitations. For instance, even when individual aptamers can be readily synthesized, elaborating them into functionalized nanoparticles (such as polymeric nanoparticles, micelles, and liposomes) tends to be tedious and expensive with low yields and difficult scale-up [18]. Recently, Urmann et al. highlighted some of these limitations and offered an insight on potential strategies to tackle these barriers [19]. Although advances in nanotechnology might overcome these constraints, additional limitations need to be addressed to allow for an efficient transition to clinical application. Key among these is the productive engagement of biological mechanisms that govern the processing of these aptamer-based delivery platforms upon internalization into target cells. These nanostructures internalize through an endocytosis mechanism (generally clathrin-mediated) and subsequently are concentrated in endocytic vesicles such as endosomes. At this stage, without any aid to escape from the endosomes, siRNA and peptide cargoes with polar and charged character and large size cannot translocate efficiently into the cytosol to exhibit their biological activity, and they instead remain entrapped in this compartment [6,20]. The amount of therapeutic cargo that can successfully access the cytosol appears to depend mainly on three factors: the number of targeted receptor molecules on the cell surface, the rates of their endocytic uptake, and the endosomal escape efficiency over the course of a cycle of internalization. Notably, some receptors should also possess a fast recycling to restore their high levels on the cell-surface and ensure continuous uptake of cargoes. As shown in Figure 1, improving any one of these levels can lead to improved net endosomal escape, greater cytosolic accessibility, and consequently to more effective biological activity.

The quantitative impacts of these factors can be illustrated by doing an admittedly over-simplified calculation. Recent studies suggest that significant siRNA-mediated gene knock-down requires approximately ~2000–5000 siRNA molecules in the cytosol [20,21]. Current estimates are that <0.01% of the internalized molecules successfully translocate into the cytosol during each cycle of internalization [20]. The majority of targetable cell-surface markers are expressed at roughly 10^4^–10^5^ copies/cell [6]. If targeting ligands carrying siRNA cargo were able to bind all of those receptors and were then all internalized, 10^4^–10^5^ of siRNAs would be delivered into endosomes during the course of one cycle of endocytosis, but only one to ten of those molecules (0.01%) would escape into the cytosol in that cycle. Given that plasma receptors typically recycle every 90 minutes for the major routes of internalization (caveolin and clathrin-mediated endocytosis), 500–5000 cycles of endocytosis (750–7500 h) would be required to deliver ~5000 siRNAs into the cytosol! In principle, the required time scale could be dramatically shortened by targeting receptors that have an expression >10^5^ (but they are very rare) with fast uptake (<20 min), increasing the escape efficiency and delivering multiple cargo molecules for each receptor binding event (e.g., micelles or lipid nanoparticles). Using this simplified framework, an improved platform carrying 10 cargo molecules that binds 10^5^ cell-surface receptors, internalizes rapidly and escapes from endosomes with an efficiency of 1% per cycle could quickly deliver 5000 siRNAs to the cytosol. In fact, many of the successful demonstrations of targeted delivery in vivo have exploited one or more of these strategies. For example, the asialoglycoprotein receptor (ASGPR) on liver cells and prostate-specific membrane antigen (PSMA) on prostate cancer cell lines are both expressed at very high levels (~10^6^ copies/cell), and internalize rapidly (15min) [20,22,23]. However, these examples are, to some extent, the low-hanging fruit of the field. Extending targeted delivery to cells that display surface proteins at more typical levels will require technological improvements. Notably, the majority of targeted receptors are expressed also on the surface of healthy cells, albeit at lower levels (~10^3^ copies/cell). Even considering an efficiency of endosomal escape of 1% per cycle, the translocation of 5000 siRNAs in the cytosol of noncancerous cells would take a considerable amount of time (~ 1 week), thus reducing the risk of off target effects.

Here, in this review, we provide a comprehensive overview of the creative approaches being adopted to generate cell targeting aptamers. We describe recent strategies that lead to the selection of nucleic acid ligands with improved targeting properties and internalization abilities. In addition, we discuss the advantages and limitations of the most promising methods utilized to improve and monitor endosomal escape of therapeutic cargoes. An exciting frontier could be to incorporate these strategies into aptamer selection protocols to identify cell targeting aptamers that ensure improved cytoplasmic accessibility of their cargoes.

## 2. In Vitro Selection of Cell Targeting Aptamers

Aptamers are generated using a selection strategy called SELEX (Systematic Evolution of Ligands by Exponential enrichment), an iterative process of exponential enrichment and amplification of desired sequences from a large combinatorial library (~10^14^ species) [24,25]. In each cycle, the evolving library is exposed to the target of interest and subjected to selection pressures that enrich nucleic acid molecules that bind the desired target. Since the invention of SELEX, a plethora of high-affinity RNA and DNA aptamers have been selected toward a wide range of different targets ranging from small molecules (i.e. metal ions, organic dyes, amino acids, and peptides) [26], to proteins [27], to complex targets (whole cells, viruses or bacteria) [15]. To date, only one aptamer (Pegaptanib, Macugen^®^) has been approved by the Food and Drug Administration in 2004 for the treatment of age-related macular degeneration (AMD) [28]. Pegaptanib is an RNA aptamer that specifically recognizes with high affinity (Kd~50 pM) and antagonizes the human vascular endothelial growth factor (VEGF_165_) [29]. Other therapeutic aptamers are currently being evaluated in various clinical trials, ranging from phase one to three. Recent reviews detail the advances of these aptamer nanomedicines in clinical trials [30,31].

Many different SELEX protocols have been employed to generate aptamers with effective cell-targeting and internalizing properties, most of which reflect two main approaches: traditional protein-based SELEX for binding to purified membrane proteins and live cell-based SELEX [32,33]. Some of the most used aptamers that target cancer antigens are listed in Table 1.

**Figure 2:**
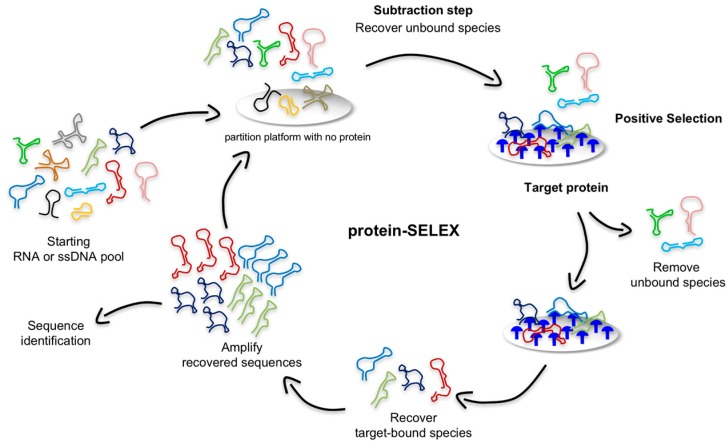
Schematic illustration of the protein-based SELEX. In a typical protocol, a subtractive step is first performed to remove nucleic acid sequences with significant affinity toward the partition platform used during the SELEX (e.g., beads, nitrocellulose filter, or chip-based approaches). The recovered unbound sequences are then incubated with the target protein (depicted in blue), and target-bound ligands are separated from the unbound population, recovered and amplified for the next SELEX round.

As shown in Figure 2, in a typical protein-based SELEX, the nucleic acid library is mixed with recombinant purified target protein, such as the soluble domain of a cell surface antigen (depicted in blue) that is overexpressed and/or mutated in diseased cells. Traditional bead-, resin-, membrane-, or chip-based partition approaches are employed to separate target-bound sequences from unbound species [50]. These strategies allow for low non-specific binding and easy control of the conditions. For example, Lupold et al. successfully selected two 2′-fluoro-modified RNA aptamers (termed A9 and A10) using a purified fusion protein containing the extracellular portion of the PSMA immobilized on magnetic beads [40].

**Figure 3:**
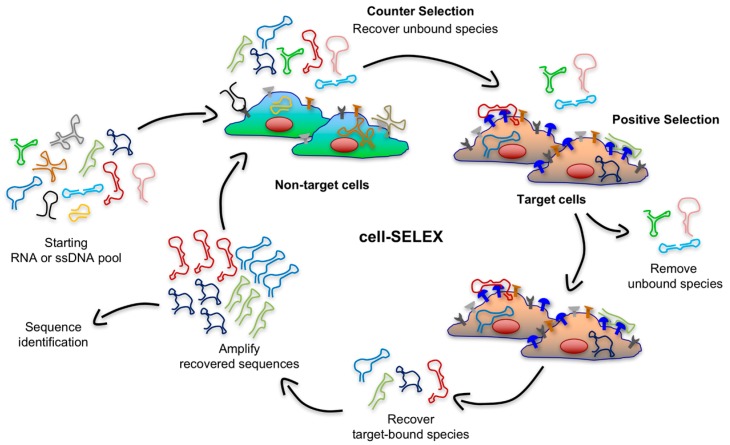
Schematic illustration of the cell-SELEX. This procedure consists of four main steps for each round of selection: (i) counter-selection by incubating the nucleic acid library with negative cells (green cells) that do not express target antigens, (ii) a positive selection by incubating recovered unbound sequences with positive cells (tan cells) expressing cell-surface antigens (depicted in blue), (iii) recovery of target-bound sequences, and finally (iv) re-amplification of recovered species.

Although, most cell targeting aptamers have been generated using protein-based selections, recombinant purified proteins may not fold into the correct 3D structure that is formed under physiologic conditions [51]. In most cases, only the extracellular domain of a cell-surface receptor is used as target during the selection and the absence of a transmembrane domain may alter the overall 3D conformation. Moreover, recombinant proteins are generated through in vitro expression systems, which often do not include the presence of post-translational modifications. Additionally, in some cases cell-surface proteins are insoluble in their recombinant forms, require interactions with other cell components (e.g., G protein-coupled receptor), or form multimeric and/or multivalent structures [52]. Thus, RNA and DNA aptamers selected through a protein-based SELEX approach might not be able to recognize the same target when embedded in a physiological milieu. For instance, the Sullenger group selected RNA aptamers against the histidine-tagged epidermal growth factor receptor (EGFR)vIII ectodomain [53]. This recombinant protein was expressed and purified using an *Escherichia coli* system, which lacks post-translational modifications. Selected aptamers were not able to bind the same protein when it was expressed in eukaryotic cells, which the authors concluded is due to the lack of one specific post-translation modification (i.e., glycosylation), significantly altering the structure of the target protein [53]. Live cell-based SELEX (or cell-SELEX) overcomes these limitations by using whole living cells as the selection target so that surface antigens are displayed in a more native environment (Figure 3). In contrast to the protein-based SELEX, cell-SELEX does not need information regarding native conformation or biological function of target proteins, and aptamers can even be generated against unknown cell-surface antigens. This method relies on the difference between the expression pattern of cell-surface receptors in target cell population (e.g., cancer cells) and the receptor pattern in control cell line (e.g., healthy cells) [35]. Thereby, a counter selection (or subtractive selection) is implemented in early rounds of the cell-SELEX to exclude sequences that have an affinity for components present on the surface of both target and non-target cells. As shown in Figure 3, during this subtractive step, the library is incubated with a control cell line and the unbound sequences are recovered and subsequently incubated with target cells.

Aptamers from cell-SELEX can be used to identify novel tumor-associated biomarkers. For example, aptamers that recognize specific tumor cells can be used as affinity capture reagents to isolate their biomolecular targets [33,54], followed by liquid chromatography-mass spectroscopy (LC-MS) for target identification [33]. Confirmation of the target identity can be further assessed by testing the cell binding properties of aptamers toward a cell line in which the expression of the target gene is silenced either via an RNAi or CRISPR-Cas9 system [55]. Cell-SELEX therefore offers tremendous advantages to generate aptamers potentially usable in clinical applications. However, it is also relatively complex and often requires more rounds of positive selection compared to protein-SELEX. In fact, the lack of knowledge about identity and expression level of the biomarker of interest might result in the enrichment of many unrelated/unwanted aptamers binding off-target surface molecules co-expressed on target cells. Therefore, more rounds of counter selection are required to improve the selectivity of aptamers.

## 3. Recent Advances in Cell-internalization SELEX

**Figure 4:**
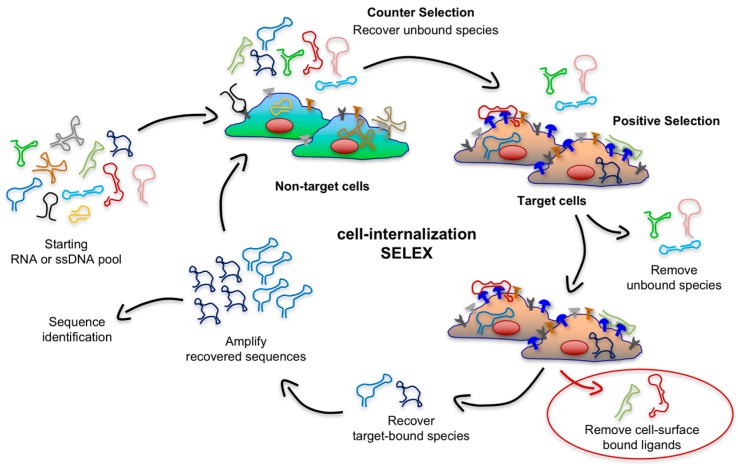
Schematic illustration of the cell-internalization SELEX. This modified selection protocol aims at selecting nucleic acid ligands that are able to both bind cell-surface antigens (depicted in blue) and their ability to rapidly internalize into the cell under physiological conditions, while effectively eliminating aptamers that lack internalizing ability and aptamers that only slowly internalize into the cells because of the target they bind.

An ideal platform for targeted delivery should possess a high rate of endocytosis upon the binding of a highly-expressed receptor. Recent years have seen the emergence of improved and creative SELEX approaches to generate targeting aptamers that rapidly internalize into target cells. These strategies go beyond simply finding aptamers that bind a given cell by depleting sequences that lack internalization ability or that are endocytosed slowly while enriching sequences that rapidly internalize upon binding with a biomarker on the surface of target cells. To accomplish this, the Giangrande group pioneered a modified cell-SELEX methodology that they termed cell-internalization SELEX, in which they introduced a stringent, high-salt wash of target cells after incubation with the RNA library [46]. As shown in Figure 4, this approach enables the recovery of nucleic acid sequences that rapidly internalize while discarding all non-internalizing cell surface binders and those that internalize with a slower rate because of the properties of the cell-surface receptor they recognize or the mode of interaction. Notably, Thiel et al. generated aptamers that were effectively endocytosed into target cells upon binding with human epidermal growth factor receptor 2 (EGFR2 or HER2), a transmembrane protein overexpressed in breast cancer cells. The fast uptake and targeting properties of these nucleic acid ligands were then exploited to deliver siRNAs targeting *Bcl2* gene in breast cancer cells [46].

Later reports highlighted that the stringent, high-salt wash alone was not sufficient to remove the totality of non-internalizing sequences bound to the cell surface. A second strategy, introduced by Levy group, exploits a cocktail of RNases to digest cell surface bound sequences. This group used aggressive nuclease treatment in several selections to generate 2′-fluoropyrimidine-containing RNA aptamers that readily internalize into various target cells [38,56]. In particular, the C1 and Otter aptamers were able to penetrate into a diverse collection of mouse and human cells, including primary cells [56]. These aptamers are generalists with limited specificity, so they are not ideal candidates for targeted delivery applications, but they could be exploited as tools to deliver siRNAs or regulatory peptides for basic research [57]. Using the same nuclease strategy to remove non-internalizing, cell-surface bound aptamers, the Levy group selected an anti-human transferrin receptor (hTfR) aptamer called c2 that rapidly internalizes in various hTfR-expressing tumor cell lines [38]. This cell-internalizing aptamer effectively delivered various molecular cargoes to living cells, such as a small chemotherapeutic drug (e.g., doxorubicin) [58], therapeutic oligonucleotides (e.g., NF-κB decoy, and microRNA-126) [58,59] and siRNA-loaded lipid nanoparticles [38]. Unfortunately, c2 competes for the binding of the receptor with its natural ligand, i.e. transferrin (Tf). Although this does not represent a limitation for in vitro applications, the high concentration of circulating Tf (ranging from 1 to 2.4 mg/ml) [60] is expected to interfere with binding of c2 to hTfR in vivo. Therefore, Maier et al. recently selected a novel aptamer, named Waz, which recognizes hTfR via a different site than Tf, thus avoiding competition for the receptor binding and potentially signaling a strong potential for the Waz aptamer in in vivo applications [39].

A third method to remove cell-surface bound aptamers is to treat the surface of cells with proteinase K after incubation with aptamers. This treatment leads to the digestion of protein domains exposed on the cell surface, including those targeted by the aptamers. Thus, the RNA ligands that are no longer bound to their cognate epitopes are simply washed off. In a recent paper, the de Franciscis group exploited this approach to select cell-internalizing aptamers that bind insulin receptors to human glioma cells (U87MG cells) [49].

An important consideration of cell-based selection protocols is that cells having a reduced membrane integrity, such as late apoptotic and necrotic cells, can indiscriminately take up nucleic acids, leading to enrichment of aptamers that non-specifically bind these cells. Partitioning methods that fail to eliminate these populations of cells drastically reduce the success rate of a selection process. To address this, the Mayer group developed what they termed fluorescent-activated cell sorting (FACS)-SELEX [44]. In this strategy, Raddatz et al. first performed a calcein staining of live cells and then used FACS to discriminate between dead and live cells within a CD19^+^ Burkitt’s lymphoma cell sample. Single-chain DNA sequences that were internalized only into the viable cells were recovered and then amplified for more rounds of selection. The authors demonstrated that a DNA aptamer named C10 [44] and a truncated form designated C10.36 [45] were able to distinguish between Burkitt’s lymphoma B cells and primary, non-malignant B cells. Both of these nucleic acid ligands were taken up into Burkitt’s lymphoma cells with fast kinetics via clathrin-mediated endocytosis. A combined strategy that utilizes key features of FACS- and cell-internalization-SELEX could represent an intriguing strategy of selection because it would simultaneously reduce limitations associated to cell-based SELEX and allow for direct monitoring of aptamer enrichment at each round of the selection via a dye-labeled RNA pool.

Certain receptors, such as GPCRs, are internalized only upon binding of their ligands in a process called ligand-induced uptake. Interestingly, targeting different epitopes on the same receptor can affect the route of endocytosis of a defined ligand. For example, Austin et al. demonstrated that an anti-EGFR monoclonal antibody and this receptor’s natural ligand, EGF, are taken up by two different mechanisms of endocytosis after binding to distinct epitopes on EGFR [61]. Aptamers that target different sites on a certain receptor could similarly experience different fates upon internalization. With this in mind, Zumrut et al. adopted a variant of cell-internalization SELEX termed Ligand-Guided Selection (LIGS) to direct aptamers to or away from a known receptor epitope [62]. LIGS exploits natural ligands or other high-affinity reagents such as monoclonal antibodies that recognize the same target receptor to displace and elute specific aptamers. Specifically, when this displacing ligand binds its cognate antigen on a certain cell-surface marker, it outcompetes and displaces aptamer sequences that interact at that site. Zumrut et al. utilized LIGS to select aptamers that bind to CD3ε–an ectodomain of T-cell Receptor (TCR)–expressed on Jurkat cells [62]. At each round of the selection, target cells were pre-treated with a partially enriched DNA pool that recognized Jurkat cells. An excess of high-affinity binding anti-CD3 antibody specific for the CD3ε domain was then co-incubated with the same target cells previously loaded with the DNA pool. This allowed displacement of aptamer sequences that bound to the same site of the antibody. Eluted sequences were then recovered and used in the following rounds of selection. In principle, the LIGS strategy could be very useful in directing aptamers to alternative epitopes of the same receptor, thereby steering the binding event into different downstream cellular outcomes.

## 4. Strategies to Enhance and Monitor Endosomal Escape of Cargoes

The therapeutic success of a given cell-internalizing platform can be strongly dependent on the ability of that platform to mediate efficient cytosolic translocation of cargoes. Thus, the efficiency of endosomal escape during a cycle of uptake needs to be improved significantly relative to that estimated for traditional delivery systems (i.e., <0.01%) [20]. Over the past years, several strategies to increase cytoplasmic accessibility of cargoes have been developed that either exploit endosomal destabilizing agents that alter permeability of endosomal membranes, such as chloroquine and other small endosomolytic molecules, or that utilize engineered viral and antimicrobial peptides with membrane-penetration properties [63]. Unfortunately, both approaches suffer from some limitations. When used at the effective concentrations, endosomolytic agents lyse not only endosomes that contain the therapeutic payload, but also a significant fraction of other vesicles including lysosomes [20]. This can lead to the release in the cytoplasm of lytic enzymes, resulting in unacceptable cell toxicity. Engineered variations of cell penetrating peptides (CPPs) (such as truncated HIV-1 TAT peptide or Aurein1,2) containing a few hydrophobic, cationic domains or a combination of them represent promising alternatives that are reported to be non-toxic in several studies [64,65,66]. However, once therapeutic cargoes are internalized into the endosomes, CPP’s can enhance their escape of only five- to eight-fold, which is still not enough to generate effective delivery platforms for clinical applications [20]. The following section highlights strategies and technologies that could be adopted into current cell-internalization SELEX protocol to enhance cytosolic translocation properties of aptamers and, consequently, of their cargoes.

### 4.1. Novel Strategies to Enhance Cytosolic Accessibility of Payloads

**Figure 5:**
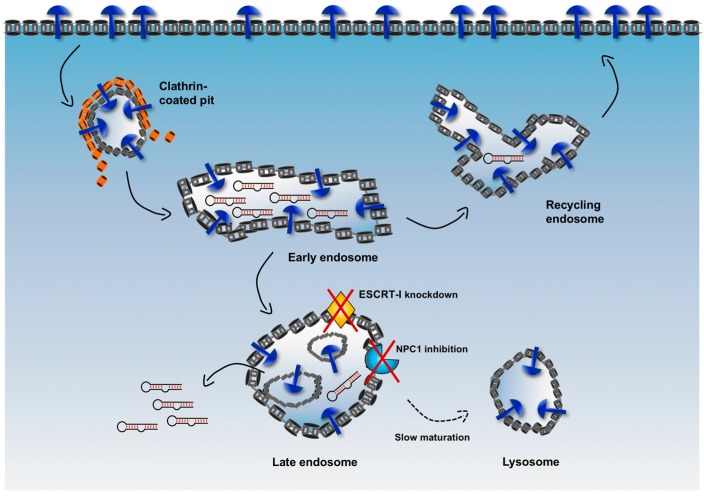
Schematic illustration of the expected vesicular trafficking of internalized aptamers and potential strategy to enhance endosomal escape. Upon binding to the target receptor, aptamers enter cells via clathrin-mediated endocytosis (or alternatively via caveolin-dependent uptake) and then are trafficked into early endosomes. In this compartment aptamers can also dissociate from the target receptor. Some receptors (such as transferrin receptor) can be recycled back to the surface, while other (like receptor tyrosine kinases, RTKs) are sorted to the endolysosomal compartment, which results in their downregulation by proteolysis. Internalized aptamers can follow a similar fate. They can be sorted back to the surface via recycling endosomes or into late endosomes. Some innovative strategies aim to delay endosomal maturation and extend the residence time of internalized molecules in non-recycling vesicles (for instance, targeting endosomal-associated proteins, such as ESCRT-I and NPC1). This can enhance the overall escape rate during each cycle of endocytosis.

A complex system of intracellular vesicles and trafficking is responsible for the fate of an internalized cargo. As shown in Figure 5, upon endocytosis, any cargo contained in endocytic vesicles is trafficked into endosomes (early endosomes), from which it can be sorted either back to the surface of the cell (recycling endosomes) or into other compartments (late endosomes) to finally reach the lysosomes where it is at risk of degradation by hydrolytic enzymes. Therefore, a major limitation for an internalized delivery platform occurs when it is sorted in vesicles that are headed to the lysosomal compartment. In fact, when this platform is located in non-recycling endosomes, there is only a narrow window of time that allows escape of cargoes into the cytosol before their degradation.

To increase the efficiency of endosomal escape, emerging strategies aim to delay endosome maturation of non-recycling vesicles, thus increasing retention of cargoes in these vesicles and avoiding fast degradation in lysosomes [67]. For example, the endosomal sorting complex required for transport-I (ESCRT-I) plays a critical role in the maturation and trafficking of endosomal cargoes, and, when ESCRT-I is associated with other accessory proteins, it is involved in the generation of particular late endosomes, i.e. multivesicular bodies (MVBs). By knocking down two components of ESCRT-I (i.e., TSG101 and VPS28), Wagenaar et al. observed that delivered antagomiR able to downregulate the oncogenic miRNA-21 displayed increased cytotoxic effect and tumor regression in vivo [68].

Similarly, Wang et al. used an inhibitor of the Niemann-Pick type C1 (NPC1) protein, termed NP3.47, to delay maturation of non-recycling endosomes such as late endosomes and lysosomes [69]. NPC1 is a late endosomal, membrane-associated protein that regulates cellular cholesterol trafficking pathways [70]. Interestingly, co-treatment of cancer cells with NP3.47 and an siRNA delivered via lipid nanoparticles (LNPs) resulted in an enhancement of both siRNA-mediated gene silencing and retention time in late endosomes/lysosomes as compared with controls in a variety of cell lines [69], potentially indicating that increasing the residence time of a cargo in non-recycling endosomes can extend the window of time for a more efficient escape into the cytosol of RNA sequences.

The cellular endosomal trafficking machinery is complex. Understanding this complexity and elucidating the roles of its major components could rationally accelerate the development of more efficient strategies for targeted delivery. For instance, as detailed above, altering the function of ESCRT-I or using NPC1 inhibitors demonstrated that effective and careful manipulation of factors involved in the cellular endosomal trafficking machinery led to more efficient and apparently non-toxic endosomal escape of therapeutic RNA cargoes. The possible implications of these results are significant. In theory, delaying maturation of non-recycling vesicles is a strategy that could be adopted in a selection process. In the current cell-internalization SELEX, upon binding to a cell-surface marker, a fraction of the internalized nucleic acid ligands is recycled back to the plasma membrane, and then endocytosed again for a new cycle of internalization/recycling. On the other hand, other aptamer sequences are expected to be sorted first into late endosomes and finally to lysosomes, where they are efficiently degraded by lysosomal nucleases, showing an overall poor endosomal escape efficiency. However, the use of a modified selection in which one or more endosomal trafficking/sorting regulators (such as ESCRT-I or NPC1) are targeted to delay endosome maturation could enhance the retention of aptamers in non-recycling vesicles. Extending the window of time before their lysosomal degradation could offer the chance of a more favorable endosomal escape of a significant amount of nucleic acid sequences.

An alternative to these strategies could be represented by the conjugation of aptamers to pH-dependent polymeric or peptide molecules that become protonated in acidic compartments. Interestingly, when located in mature endosomes (pH ≤ 6), these molecules can trigger an osmotic swelling of the vesicles (i.e., proton sponge effect), which enhances cargo release into the cytosol. An example of this approach was reported by Liu et al. [71]. These authors conjugated an anti-PSMA aptamer-siRNA chimera via a dsRNA binding domain (dsRBD) to a pH-dependent polyhistidine. The presence of this short histidine oligomer enhanced cytosolic delivery of the aptamer chimera, leading to improved siRNA-mediated silencing of its target gene [71]. Further in vitro and in vivo studies are required to fully assess safety and efficacy of this strategy.

An example of an aptamer that can, in some extent, gain cytosolic accessibility is the DNA aptamer (AS1411). This molecule is a G-quadruplex forming oligonucleotide that specifically binds nucleolin, a multifunctional nuclear ribonucleoprotein overexpressed in cancer cells where it shuttles among the nucleus, the cytoplasm, and the cell-surface. AS1411 was originally identified during a screen of antisense oligonucleotides for antiproliferative activity toward several human tumor cells [72]. This DNA aptamer is currently undergoing clinical trials as anticancer agent [73]. Interestingly, AS1411 can internalize via macropinocytosis, but a recent work demonstrated that its initial cell uptake is independent of binding to nucleolin [74]. Interaction with this ribonucleoprotein, however, is still essential for the antiproliferative activity of AS1411 and to stimulate further uptake of AS1411 via macropinocytosis [75]. The mechanism by which AS1411 escapes from the macropinosomes/endosomes and accesses the cytoplasm remains unclear, but it is likely that nucleolin’s ability to shuttle between different compartments seems to play an essential role.

### 4.2. Traditional and Innovative Methods to Monitor Endosomal Escape of Cargoes

Generating efficient cytoplasmic delivery platforms requires highly sensitive and robust methods to measure cytosolic penetration and discriminate between internalization and cytosolic accessibility. Improvements upon current methodologies are needed to achieve this distinction and to evaluate aptamer uptake during the SELEX process. Fluorescence microscopy is one of the most used strategies to assess internalization of dye-labeled aptamers in target cells. Standard microscopy techniques enable qualitative analysis of the aptamer-mediated intracellular delivery of molecular payloads, especially when dual-labeled nanostructures are used, bearing one fluorophore on the targeting ligand and a different dye on the cargo [58,76]. Upon receptor-mediated or ligand-induced endocytosis, the internalized aptamers and cargoes are located in endocytic vesicles, where their increased local concentration leads to a higher signal-to-noise ratio and punctate appearance. Unfortunately, it is more challenging to discern aptamer fluorescence in the cytoplasm using conventional microscopy settings and instrumentation. Dispersion of nucleic acid molecules into the cytosol decreases overall aptamer concentration and drastically reduces the signal-to-noise ratio. Therefore, novel strategies are needed to address this limitation. This section analyzes current methods used for assessing internalization and endosomal escape of different molecular cargoes. Our goal is to highlight advantages and drawbacks of these methods and identify those that could be incorporated into a SELEX process and be part of the aptamer technology.

Intriguing results were recently provided by the Giangrande group. To visualize internalized aptamers, Hernandez et al. developed a method called antibody amplification microscopy [77]. In brief, an RNA aptamer (C4-3) that internalizes into TrkB-expressing cells was first conjugated to fluorescein (FAM), but the conjugate gave only a minimal fluorescence upon cell incubation with FAM-C4-3. The authors then performed immunostaining using a primary antibody that recognizes FAM and a dye-labeled secondary antibody labeled with multiple AF488 fluorophores per molecule, thus increasing the overall brightness of this reagent. Antibody-mediated amplification of the fluorescence signal allowed effective detection of FAM-C4-3 inside TrkB-expressing cells. Although this strategy did not discriminate between cytoplasmic and endosomal signal of the aptamers, it suggests that an amplification-based approach could potentially be used to detect aptamers in the cytosol. Variations of this method able to specifically target cytoplasmic RNA molecules might represent a valid alternative for aptamer detection.

Exploiting a novel and highly sensitive fluorescence microscopy technique, a recent study showed release of siRNA delivered via lipoplexes (i.e. lipofectamine-siRNA complexes) or LNPs from individual endosomes [21]. Fluorescence of siRNA molecules was monitored using an imaging system based on multiple focal planes with different exposure times. Long exposure times were used for one of these focal planes to intentionally overexpose bright areas (i.e., endocytic vesicles containing lipoplex-siRNA complexes) and detect the weak fluorescence of free siRNAs translocated in the cytosol. This strategy could potentially have a great impact on understanding and monitoring endosomal escape of cargoes. However, the unconventional microscopy instrumentation is not accessible to the majority of research laboratories, and its success is dependent on the use of sophisticated approaches. Therefore, while it is starting to become possible to discern translocation of cargoes from endosomes to the cytoplasm using fluorescence microscopy, additional developments are needed. Moreover, many microscopy strategies are often time consuming especially if several probes or incubation steps are required, as in the case of the antibody amplification microscopy method above. In general, the low-throughput nature of microscopy does not make this technology the ideal strategy to monitor acquisition of a certain phenotype (such as internalization or cytoplasmic translocation) of a large RNA pool during the course of a SELEX process, where high-throughput systems are preferred.

An attractive high-throughput approach used to assess both binding and internalization properties of fluorescently labeled aptamers is flow cytometry. In this case, to discriminate between surface bound aptamers and internalized species, a stringent, high-salt washing or an enzymatic treatment is required (such as using a cocktail of RNase or Proteinase K as described above). Therefore, the efficient removal of these surface-bound species plays a critical role in assessing aptamer endocytosis. Although flow cytometry is a high-throughput approach, it suffers from some of the same limitations as fluorescence microscopy, in that this method is not able to discern between cytoplasmic and endosomal localization of aptamers.

The Dehua Pei group offered a potential solution that might overcome the difficulty in monitoring molecules that have escaped from endosomes. In a recent paper, Qian et al. exploited a pH-sensitive fluorophore called naphthofluorescein (NF) coupled to peptide sequences to assess their cytoplasmic translocation [78]. Interestingly, only the deprotonated form of NF is fluorescent (pKa of NF is ~7.8). Therefore, NF is expected to be nearly completely protonated and non-fluorescent inside the acidic endosomes (pH ≤ 6.0), but a fluorescence enhancement could be detected by flow cytometry or fluorescence confocal microscopy, indicating cytosolic accessibility (pH 7.4). The Pei lab took advantage of these NF properties to screen and compare endosomal escape efficiencies of several CPPs. First, the authors assessed and compared the internalization properties of every peptide using a pH-insensitive dye (i.e., rhodamine). Then, the endosomal escape of NF-CPP conjugates was monitored by flow cytometry. Notably, only a minimal fluorescence was observed when NF was conjugated to control peptide sequences that are not able to translocate into the cytosol. In contrast, the authors detected significant fluorescence enhancement upon internalization of NF-CPP conjugates. The extent of fluorescence activation was directly correlated with the endosomal escape properties of each peptide sequence. Using this strategy, the authors identified a cyclic peptide (called cFΦR4) that escapes from the endocytic pathway ~5-fold more efficiently relative to the well-characterized HIV-1 TAT peptide.

Conjugating pH-sensitive fluorophores to known cell-internalizing aptamers could represent a promising and accessible strategy to compare both internalization and cytosolic translocation properties of sequences identified in previous selections. Moreover, a modified cell-SELEX that uses a nucleic acid library linked to a pH-sensitive dye could be used in each round of the protocol to monitor cytosolic entry via flow cytometry. This selection approach could be further modified by adopting subcellular fractionation as a partition method to separate aptamer sequences located in different subcellular compartments (such as cytosol and endocytic vesicles).

Arguably, highly efficient methods to evaluate productive cytosolic translocation of aptamers and/or their associated cargoes should directly correlate the escape efficiency with a functional readout, such as a biological output that is only activated by internalized payloads that effectively access the cytosol. However, the biological activities for most therapeutic cargoes can only be monitored over time using a loss-of-function approach, such as target down-regulation by RNAi, and they often require a time scale (usually >12 h) that is not compatible with a SELEX protocol due to accumulated digestion of internalized aptamers by lysosomal or cytosolic nucleases. Typically, validation of the biological activity of therapeutic cargoes delivered via aptamers can only be evaluated after some rounds or at the end of the selection. In contrast, a different functional readout that exploits rapid gain-of-function assays, such as monitoring increase of fluorescence or luminescence as a consequence of a biological effect, could be more easily incorporated in a SELEX protocol.

Recently, Lonn et al. have developed a fluorescence-based quantitative cytosolic translocation assay to monitor peptide-mediated delivery of cargoes into the cytosol [65]. They reported a split-GFP complementation assay in which a large non-fluorescent fragment of GFP, composed of 10 out to the 11 β-strands of GFP, was expressed in cells. The complementary component (GFPβ-11) required for the function (i.e. fluorescence) was delivered using cell-penetrating peptides, such as HIV-1 TAT peptide. In this approach, fluorescence can only be observed when the TAT-GFPβ-11 complex is able to escape from endosomes and interact with the non-fluorescent GFP in the cytosol. Those authors employed a set of CPPs reported to have cytosolic translocation properties and successfully restored GFP fluorescence. Importantly, the strength of the GFP fluorescence signal was used to quantitatively assess the endosomal escape abilities of each CPP, thus allowing distinction of these peptides into different classes based on their cytosolic translocation efficiency [65]. Overall, the Split-GFP complementary assay offers a sensitive assay monitoring a biological activity (protein-protein interaction) with low to zero false positives when utilized to evaluate endosomal escape properties. However, to implement this strategy in a SELEX method or to assess escape of known cell-internalizing aptamers, conjugation to the GFPβ-11 motif should ensure retaining of aptamer folding and restoring of GFP fluorescence. Therefore, to minimize inter- and intra-molecular interactions, different coupling strategies should be evaluated before starting a selection.

An intriguing alternative to this approach might exploit a split-aptamer system that generates a specific readout only upon formation of a fully-functional aptamer structure. One component of this split system could be directly connected to a combinatorial library, and the other component could be expressed in the target cells, such that signal is only observed upon successful delivery of the complementary strand. In this regard, our lab recently introduced a tool to monitor RNA-RNA interaction in bacteria. In brief, taking advantages of the light-up properties of fluorogenic RNA aptamers, we engineered a split version of the broccoli RNA aptamer [79]. Hybridization of two non-fluorescent RNA strands restores Broccoli fluorescence in living cells. Current efforts in our lab to monitor endosomal escape of aptamers are directed to express in mammalian cells one of the two strands of the Split-Broccoli system while delivering its complementary strand using known cell-internalizing aptamers. Compared to the Split-GFP complimentary assay, our approach exploits an “all-RNA system” thus offering significant advantages in terms of ease of design, synthesis, and folding assessment.

## 5. Conclusions

Over the last decade, aptamer-mediated targeted delivery of therapeutics in malignant cells has emerged as a promising cancer therapy approach with limited off-target effects. Current efforts are directed towards increasing the performance of these therapeutic aptamer nanostructures to facilitate their clinical translation. Particularly, several strategies aim to enhance the cytosolic accessibility of cargoes upon their aptamer-mediated internalization. Since its discovery, the SELEX process has undergone a continuous evolution. Existing methods have been modified and consequently evolved to meet new biological and technological requirements and ultimately to generate more versatile and potent nucleic acid ligands. In the case of cell targeting aptamers, early SELEX protocols aimed to identify nucleic acid ligands that bind recombinant purified cell-surface proteins. The evolution of this approach led to more complex selection strategies. In the early days, aptamers able to recognize whole tumor cells with high selectivity were generated through an innovative cell-SELEX method. Later, cell-internalization SELEX was introduced to select aptamers that not only bind cell-surface markers in their physiological environment but that also internalize into malignant cells. In each of these SELEX variations, additional properties, such as recognition of whole cells instead of recombinant proteins and rapid endocytosis in cancer cells, were included in the selected ligands. However, clinical translation is still limited by escape into the cytosol. Thereby, to overcome current limitations of the aptamer-mediated targeted delivery of therapeutics, a further evolution of SELEX is needed. Innovative selection protocols that incorporate technologies and tools developed for other biomolecules (such as peptides and proteins) could result in the effective identification of nucleic acid ligands with extended biological functionalities. New generations of aptamers will go beyond binding and internalization, and will be able to perform productive cytosolic translocation of therapeutics, thus accelerating clinical translation of cell targeting aptamer nanostructures.

## Figures and Tables

**Table 1:** Aptamers that bind cell-surface markers selected by protein-SELEX or cell-SELEX.

Aptamer Library	Name of the Aptamer	Biomarker	Type of SELEX	References
2′F-RNA	E07	EGFR	Protein-SELEX	[34]
DNA	Sgc8	PTK7	Cell-SELEX	[35]
Thio-DNA	TA1-TA6	CD44	Protein-SELEX	[36,37]
2′F-RNA	c2, Waz	CD71 (hTfR)	Hybrid SELEX ^1^	[38,39]
2′F-RNA	A9, A10	PSMA	Protein-SELEX	[40]
DNA	AS1411	Nucleolin	screening of G-rich oligos in cell lines	[41,42,43]
DNA	C10, C10.36	CD19 (+) Burkitt lymphoma	Cell-SELEX	[44,45]
2′F-RNA	B1, C1, E1	HER2	Cell-SELEX	[46]
RNA, DNA	FB4, GS24 (DW4)	mTfR	Protein-SELEX	[47,48]
2′F-RNA	GL56	Insulin receptor	Cell-SELEX	[49]

^1^ Hybrid SELEX = combination of protein- and cell-based SELEX.
